# Development of Microscopic Techniques for the Visualization of Plant–Root-Knot Nematode Interaction

**DOI:** 10.3390/plants11091165

**Published:** 2022-04-26

**Authors:** Helena Vernet, Aïda Magdalena Fullana, Francisco Javier Sorribas, Emilio J. Gualda

**Affiliations:** Department of Agri-Food Engineering and Biotechnology, Universitat Politècnica de Catalunya, Esteve Terradas 8, 08860 Castelldefels, Spain; helena.vernet@gmail.com (H.V.); aida.magdalena.fullana@upc.edu (A.M.F.)

**Keywords:** plant 3D imaging, root-knot nematode, tissue clearing, light sheet fluorescence microscopy, optical projection tomography

## Abstract

Plant-parasitic nematodes are a significant cause of yield losses and food security issues. Specifically, nematodes of the genus *Meloidogyne* can cause significant production losses in horticultural crops around the world. Understanding the mechanisms of the ever-changing physiology of plant roots by imaging the galls induced by nematodes could provide a great insight into their control. However, infected roots are unsuitable for light microscopy investigation due to the opacity of plant tissues. Thus, samples must be cleared to visualize the interior of whole plants in order to make them transparent using clearing agents. This work aims to identify which clearing protocol and microscopy system is the most appropriate to obtain 3D images of tomato cv. Durinta and eggplant cv. Cristal samples infected with *Meloidogyne incognita* to visualize and study the root–nematode interaction. To that extent, two clearing solutions (BABB and ECi), combined with three different dehydration solvents (ethanol, methanol and 1-propanol), are tested. In addition, the advantages and disadvantages of alternative imaging techniques to confocal microscopy are analyzed by employing an experimental custom-made setup that combines two microscopic techniques, light sheet fluorescence microscopy and optical projection tomography, on a single instrument.

## 1. Introduction

Among all phytopathogens, plant-parasitic nematodes are an important cause of yield losses and food security issues, especially, those of the genus *Meloidogyne*. Four out of the more than 100 root-knot nematode species (RKN) described until now are responsible for the majority of crop yield losses attributed to this genus, namely *M. arenaria*, *M. incognita*, *M. javanica* and *M. hapla*. These species are widely distributed around the world; they can parasitize a large number of plant species and reproduce parthenogenetically [1]. *Meloidogyne* spp. are sedentary endoparasitic nematodes that affect the underground organs of plants, mainly the roots. The modifications they cause in the tissue give rise to the formation of galls, limiting the absorption of water and nutrients by the plant, which ends up causing nutritional deficiencies and can even kill the plant. RKN are the most devasting plant-parasitic nematodes of vegetable crops under protected cultivation where both environmental and agronomical conditions favor the exponential growth of the nematode population densities, seriously affecting the viability and productivity of these crops [2]. Maximum vegetable crop yield losses estimated under protected cultivation in the Mediterranean basin varied from about 36% in watermelon and zucchini-squash [3,4] to 80% in melon [5]. Despite the existence of several control methods, plant resistance is the most effective and safest for the environment and health [6]. Resistant plants inhibit nematode infection, development and/or reproduction [7]. Phenotyping evaluation of the level of resistance of a given plant germplasm is usually performed by bioassays conducted to determine the number of egg masses (successful infection and development), the number of eggs (successful reproduction) and the number of eggs per egg mass (female fecundity; female reproductive success) in comparison to a susceptible standard. This procedure requires several weeks from nematode inoculation to data collection and analysis. Another way to study the level of resistance is by histopathological studies in order to know if a given plant–nematode interaction is compatible or not. In a compatible interaction, the infective second-stage juvenile (J2) penetrates the root and moves intercellularly to reach the vascular cylinder. Afterwards, secretions from the salivary glands of the nematode are injected into selected root cells, leading to their transformation into hypertrophied multinucleate cells known as giant cells (GC). Once they have established their feeding sites, the J2 becomes sedentary, and after three molts, it reaches the adult stage. If conditions are favorable, the J2 will evolve into a female with a rounded shape, which will lay the eggs in a gelatinous matrix that is found on the root surface or inside the galls. Under unfavorable conditions, the juvenile will evolve to a male that migrates out of the plant [8]. Conversely, if the reaction is incompatible, some plant-defense mechanisms can act, affecting some or several steps of the disease cycle such as the lack of induction or abnormal GC formation and hypersensitive response among others, which can be observed by microscopy. 

However, due to the opacity of plant tissue, infected roots are not amenable for light microscopy investigation without using tissue clearing techniques, which allow equilibration of the refractive index (RI) throughout a sample to reduce inhomogeneities in light scatter [9]. Tissue clearing techniques make it possible to image three-dimensional structures deep inside a sample (tomography) without the need for historical sectioning, which is particularly important for structures that are not confined within single imaging planes [10]. There are three principal tissue clearing approaches: solvent-based clearing, aqueous-based clearing and hydrogel embedding [11]. Solvent-based clearing techniques are the simplest and cheapest ones. They are comprised of two steps: (1) dehydration with lipid solvation in a series of alcohol–water mixtures with progressively less water concentration and (2) clearing by refractive index matching. Several solvents have been tested for use in either the dehydration or clearing steps. Most commonly, dehydration is now performed using methanol [12], ethanol [5,13] or 1-propanol [14]. The dehydration step is followed by a second agent that solvates additional lipid and intercalates homogeneously throughout the sample to clear it by matching the higher refractive index of the defatted and dehydrated tissue [11]. 

Benzyl alcohol/benzyl benzoate (BABB) [15], which has an RI of 1.55, is commonly used as a clearing solution because it works on many different tissue types. However, BABB is a severely toxic and corrosive substance, and produces substantial tissue shrinkage during dehydration, up to 50% [12]. To overcome these limitations, a new solvent-based clearing solution has been recently developed [13,14]. Ethyl cinnamate, or simply ECi (ethyl-3-phenyl prop-2-enoate), is an excellent clearing reagent with a refractive index of 1.558. ECi has been a Food and Drug Administration-approved food flavor and additive for cosmetic products since 2007 [16], and it is considered harmless according to the European directive 67/548/EEC.

Compared with previously reported protocols, ECi presents several advantages. It allows visualization of root–nematode interaction without the tedious work of vibratome sectioning [17], providing in a simple way 3D information of the entire gall structure; it requires less incubation time than other clearing protocols such as ClearSee [18]; it allows clarifying thicker roots than *Arabidopsis* ones [19]; it is not toxic and corrosive in the same way as BABB. As well as its non-toxicity, another advantage of ECi-based protocols is that the solution consists of only one compound, whereas BABB consists of a mixture of one part benzyl alcohol and two parts benzyl benzoate. For those reasons, there is no need to take extra care ensuring proper mixture along the clearing process through sample agitation, wearing protective equipment when handling samples or providing room ventilation during image acquisition, as happens when using BABB.

Once cleared, there are several microscopy techniques for 3D imaging of thick biological samples. For many years, the gold standard of 3D microscopy has been confocal laser scanning microscopy (CLSM) [20]. Although CLSM provides high-resolution images, it has been proved slow, time-consuming and may produce photo-bleaching and photo-damage in the sample. In addition, due to sample mounting on microscopy slides, CLSM only allows a partial view of the sample to be obtained.

During the last years, alternatives microscopy techniques for 3D imaging have been developed, such as light sheet fluorescence microscopy (LSFM) [21] or optical projection tomography (OPT) [22], overcoming some of the drawbacks of confocal microscopy. In LSFM, a plane of illumination can be generated by a laser and a cylindrical lens. Then the specimen is scanned by moving the sample in relation to a static light sheet in order to produce optical sections through its volume [23]. More interestingly, this technique permits rotation of the sample and visualization of the sample from different views, something that cannot be done with confocal microscopy. In OPT, a series of 2D projections, obtained from different angles, are acquired and the 3D structure of the tissue is generated as a stack of cross-sectional image slices using a reconstruction algorithm, commonly a filtered back-projection [24]. Both techniques, LSFM and OPT, have been successfully used to image plant tissues [25,26,27].

The goal of this work was to fine-tune clearing and microscopic methods to facilitate deep-tissue imaging of the roots in plants infected with *Meloidogyne* in order to visualize, with 3D capabilities, the degree of RKN infection and root–nematode interaction. Specifically, we will determine whether reducing the differences in refractive indices within the tissue and removing colored tissue components improve the optical transparency of biological samples. Previous works [5] used ethanol–BABB clearing and confocal imaging to study *M. incognita* in tomato cv. Durinta and melon cv. Paloma samples. Here, another simple, cheap and overall non-toxic alternative clearing solution (such as ECi) in use with different solvents (1-propanol, ethanol and methanol) is investigated, simplifying the sample handling. From the technical point of view, we will analyze the pros and cons of alternative microscopic confocal techniques to. In that sense, we have designed a setup that combines LSFM and OPT in a single instrument, thus providing new, fast and cost effective phenotyping techniques adapted to the study of cleared tomato and eggplant roots with RKN infection.

## 2. Results

### 2.1. Dehydration and Clearing 

**Figure 1:**
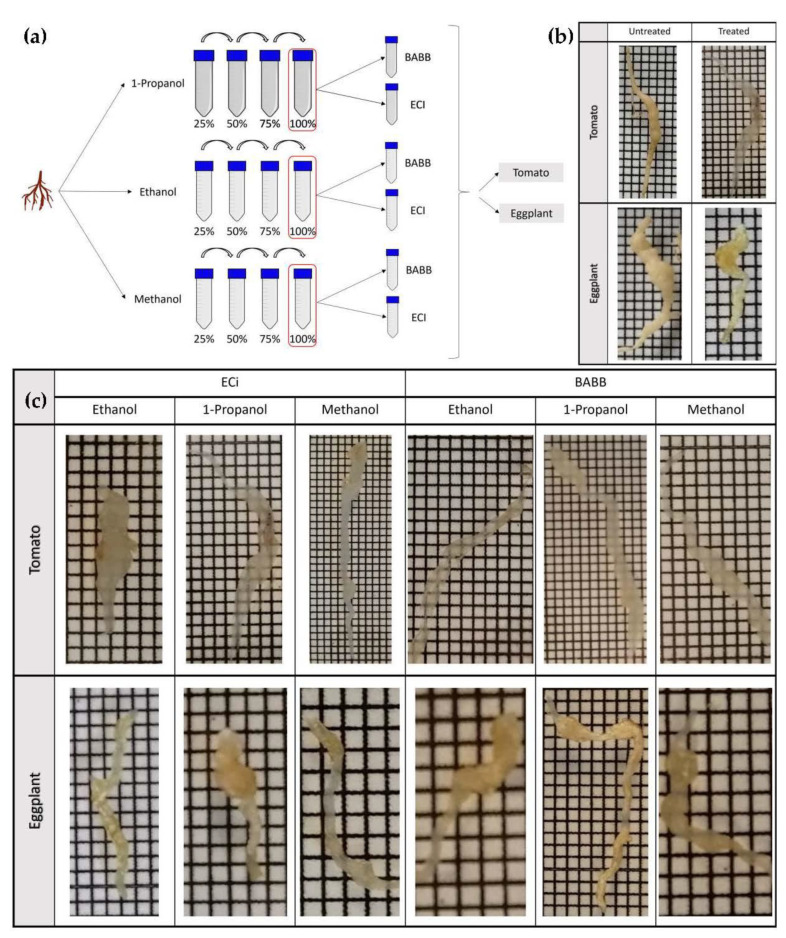
(**a**) Schematic representation of the procedure followed for the clarification of root samples. (**b**) Optical clearing of different samples of tomato and eggplant roots infected by *M. incognita* 6 weeks after nematode inoculation using a combination of 1-propanol and ECi as a dehydrating agent and clearing reagent, respectively. Photographs were taken before and after treatment with a clearing solution. (**c**) Comparison of the clearing efficiency in tomato and eggplant samples after treatment. Grid scale: 1 mm.

Clearing efficacy was assessed in root-derived samples from tomato cv. Durinta and eggplant cv. Cristal, treated with different dehydration and clearing agents. To obtain the best dehydration-clearing agent combination to achieve the desired degree of transparency for microscope imaging, a total of 12 tests were performed: 6 for tomatoes and 6 for eggplant, where 3 different dehydration agents (1-propanol, ethanol, or methanol) were combined with 2 different clearing agents (BABB or ECi) (Figure 1a).

After 24 h treatment, we proved that the formulations developed for this study were effective at clearing roots. Dehydration and clearing solutions substantially increased root transparency compared with untreated roots (Figure 1b) and grid lines were clearly observed. However, tomato and eggplant showed different behavior. Eggplant roots appeared more yellowish than tomato ones, suggesting the existence of pigments that prevent increased clearing Figure 1c. Regarding the clearing efficiency of several alcohols, it was found that the dehydration of tomato roots using 1-propanol–ECi allowed the best results to be obtained. In the case of BABB clarification, the most favorable combination for tomato was achieved using ethanol as the dehydrating agent. In eggplants, it seems that ECi performed better than BABB, although the levels of transparency were in any case worse than in tomato roots. From the pictures, the best results were achieved with methanol–ECi, ethanol–ECi and methanol–BABB combinations.

### 2.2. LSFM Imaging of Cleared Roots Galls

Cleared tomato and eggplant samples were observed under the custom-made LSFM system to determine in a more precise way the efficacy of the different clearing protocols tested, the degree of remaining scattering of the samples and the penetration range of laser light. The schematic diagram of the designed equipment, combining light sheet fluorescence microscope (LSFM) and optical projection tomography (OPT), is shown in Appendix A. 

**Figure 2:**
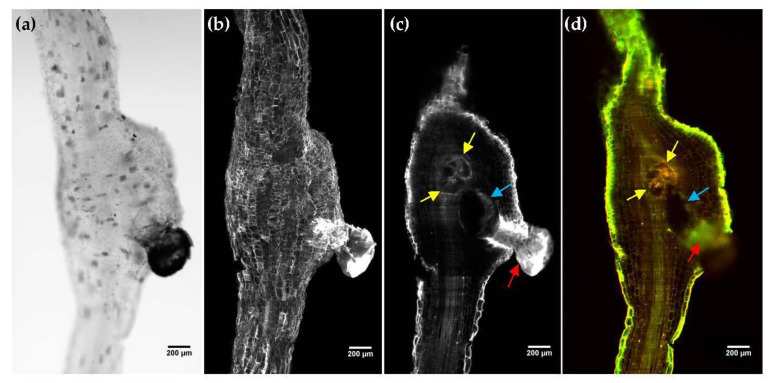
Light sheet fluorescence microscopy imaging of tomato root galls induced by *M. incognita* cleared with 1-propanol–ECi protocol. (**a**) Brightfield image. (**b**) Maximum intensity projection. (**c**) Cross-section of gall where giant cells (yellow arrows), *M. incognita* female (blue arrow) and eggs mass (red arrow) can be distinguished. (**d**) Composite image with two colors was obtained using a 525/45 nm (green) and 650/LP (red) filter. Scale bar: 200 µm.

Brightfield images (Figure 2a), obtained by trans-illuminating the sample with an LED source, allowed distinguishing regions with increased light absorption, such as pigmentation along with the tissue or the egg mass, extended out of the root gall. A close look into the interior of the sample revealed the presence of the giant cells and the female nematode. However, it was not properly resolved. To solve that, 3D LSFM imaging of roots galls was performed through the entire depth of the sample with a 3 μm step. Auto-fluorescence was excited with a 488 nm laser and measured using a 561/LP nm filter. A strong auto-fluorescence signal was detected on the egg mass (red arrow) and the epithelial cells of the root (Figure 2b). The internal structure of the roots (Figure 2c and Video S1) was also visible, allowing the individual root cells and the vascular system to be distinguished. The walls and the multiple nuclei of the giant cells (yellow arrows) were also clearly visible, as well as the female nematode (blue arrow).

In order to gain a contrast mechanism that helps nematode and giant cells identification, we also recorded auto-fluorescence with two different emission filters, a green filter (525/45 nm) and a red long pass filter (650/LP). By their combination, the radiometric contributions of the different sample compounds were highlighted (Figure 2d and Video S2). Although the resulting images did not show significant structural differences between the two filters, it appears that shorter auto-fluorescence wavelengths contributed more to the exterior of the root and the vascular cylinder (green), while giant cells and eggs mass auto-fluorescence was higher at longer wavelengths (red). The female nematode was also perfectly observed within the root sample.

### 2.3. LSFM Imaging of Cleared Tomato and Eggplant Roots Galls

Once we had demonstrated the LSFM system performance for nematode–root interaction, we evaluated the efficacy of the different clearing protocol tested in Section 2.1. Full-thickness cross-sectional optical images of the cleared root gall samples were generated from the z-stack using the “orthogonal projections” plugin in FIJI software [28]. Of particular interest in assessing the clearing efficiency, the XZ cross-section allowed visualizing simultaneously the external cell layer and internal plant tissue structures, and allowed determining the image quality of the deepest regions of the root gall. 

**Figure 3:**
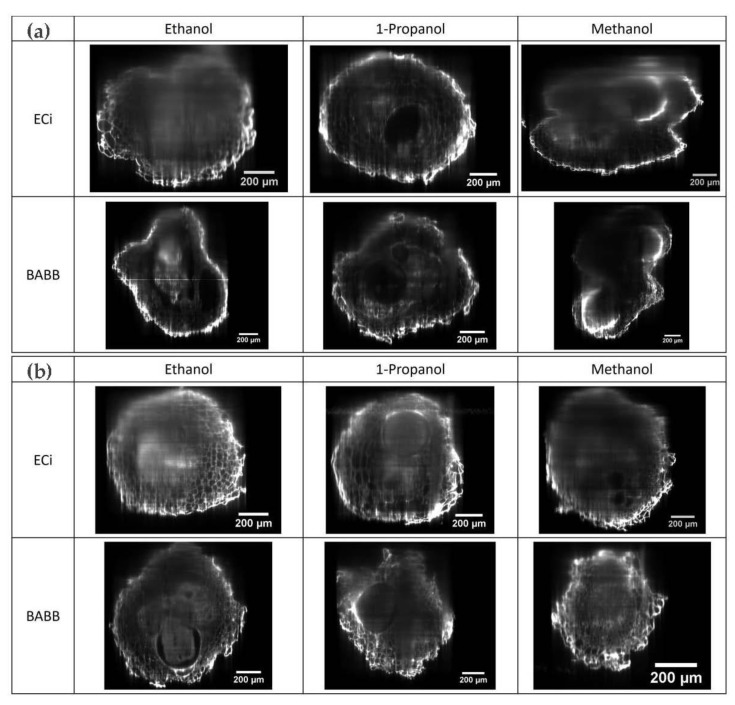
Comparative study of LSFM penetration depth, for different dehydration (ethanol, 1-propanol and methanol) and clearing agents (ECi and BABB), in (**a**) tomato and (**b**) eggplant galled root samples. The cross-section along the XZ plane allows comparing and evaluating the degree of penetration and image quality distortion obtained in each clearing protocol. The lower part of the images corresponds to the closest to the camera. Scale bar: 200 µm.

It can be seen that ECi enabled the imaging of galls throughout the entire thickness of the root gall, at least with a similar degree of transparency than BABB-cleared samples, but without the associated drawbacks in terms of toxicity and corrosion. In particular, we observed that the optimum images in tomato samples (Figure 3a) were obtained with the combination of 1-propanol–ECi-treated samples (Video S2), while ethanol–ECi and methanol–ECi did not allow the deepest regions to be properly observed. Ethanol–BABB (Video S3) and, in particular, 1-propanol–BABB (Video S4) clarification were also valid combinations for tomato root samples. 

In contrast to the observations through photography shown in Figure 1, for the eggplant samples (Figure 3b), a high degree of transparency was achieved, allowing samples to be observed along more than half of their depth. It appears that the best clarification combination was ethanol–BABB (Video S5), although 1-propanol–ECi (Video S6) also rendered good results. 

**Figure 4:**
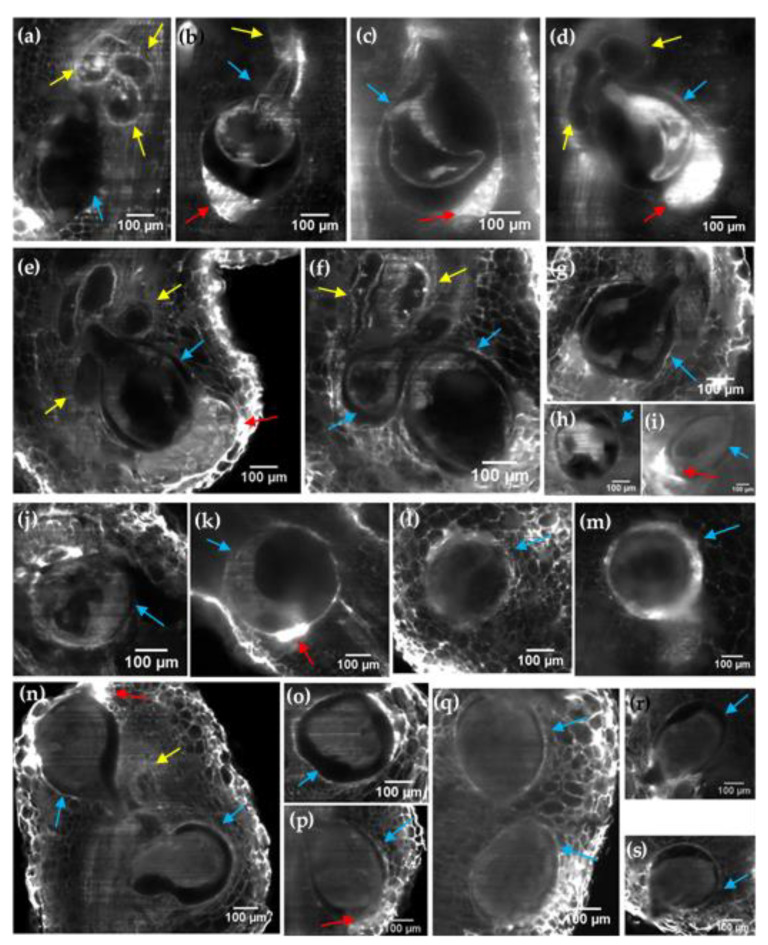
LSFM imaging of tomato (**a**–**m**) and eggplant (**n**–**s**) galled roots 6 weeks after *M. incognita* inoculation. (**a**) Tomato gall roots 1-propanol–ECi treated. (**b**–**d**) Tomato gall roots methanol–ECi treated. (**e**–**i**) Tomato gall roots 1-propanol–BABB treated. (**j**) Tomato gall roots ethanol–BABB treated. (**k**–**m**) Tomato gall roots methanol–BABB treated. (**n**,**o**) Eggplant gall roots ethanol–BABB treated. (**p**,**q**) Eggplant gall roots 1-propanol–ECi treated. (**r**,**s**) Eggplant gall roots 1-propanol–BABB treated. Giant cells (yellow arrows), nematode females (blue arrows) and egg masses (red arrows) are indicated. Scale bar: 100 µm.

LSFM makes it possible to observe in detail the structures induced by the nematode in tomato and eggplant roots (Figure 4). The characteristic shape of the adult female can be differentiated (blue arrows), as well as certain internal structures, such as the cuticle of the female nematode. The internal structure of the giant cells was also visible, allowing the multiple induced nuclei to be distinguished (Figure 4a,b,f,n). We also noticed that some giant cells lacked metabolic activity with no apparent nuclei (Figure 4d,e). A strong auto-fluorescence signal was detected on the egg masses (red arrows in Figure 4b–e,i,k,n,p), allowing the observation of individual eggs (Video S4). We also observed that larger egg masses with a greater number of eggs were produced in tomato (Video S7) than in eggplant (Video S8). 

### 2.4. OPT Imaging of Cleared Tomato Root Galls

As an alternative to LSFM, we also tested optical projection tomography (OPT) as a tool to provide information on the shape and structure of root–nematode interactions. The OPT principle relies on the rotation of a sample to acquire 2D projections at different angles. These acquired projections are sufficient to recover an accurate 3D reconstruction of the sample structure applying back-projection algorithms. Since LSFM and OPT share a similar architecture, our setup can be easily converted into an OPT imaging system [29,30,31]. The only requisites for OPT imaging are that the entire sample must be well centered along the rotation axis and all its thickness must be in focus. However, microscopy objectives normally have a short depth of field (DoF). To solve that point we added an iris diaphragm in the detection path in order to extend the objective DoF. This aperture permits a larger fraction of the sample to be kept in focus at the expense of reducing the amount of light that reaches the camera.

**Figure 5:**
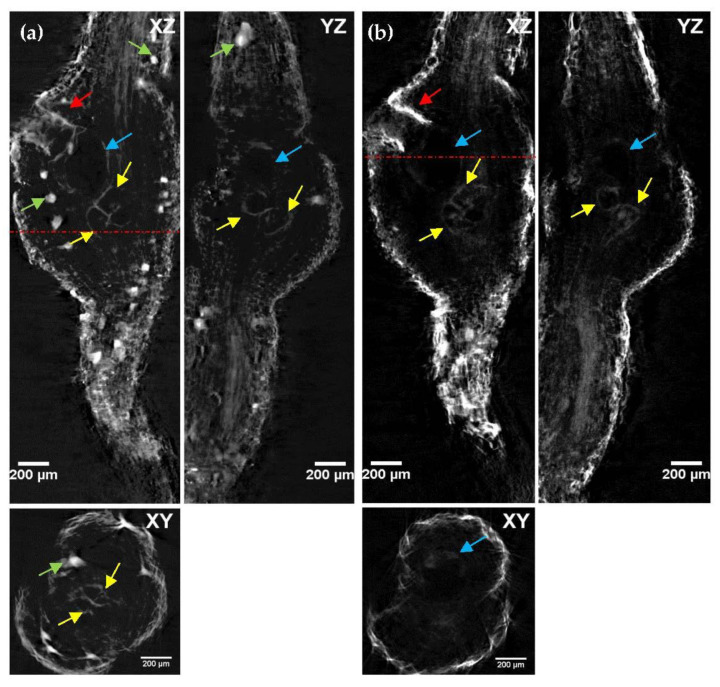
Optical projection tomography orthogonal views of a tomato root infected by *M. incognita* 6 weeks after nematode inoculation and cleared with 1-propanol–ECi acquired in (**a**) brightfield OPT mode and (**b**) fluorescence OPT mode. Giant cells (yellow arrows), adult female (blue arrows) and egg mass (red arrows) can be distinguished. In OPT brightfield mode, pigmented cells are also visible (green arrows). Scale bar: 200 µm.

Two different OPT modalities were tested in 1propanol–ECi-cleared tomato roots: brightfield and fluorescence. For brightfield OPT, the sample is trans-illuminated with an LED lighting system and the light absorption pattern is captured by the camera as it rotates (Video S9). For fluorescence OPT, the same light sheet used in LSFM excites sample auto-fluorescence, but the cylindrical lens is defocused, to generate a thicker sheet of light, illuminating the whole sample. In both cases, once centered and focused, the sample is rotated through 360° with 0.5° steps using a stepper motor, and 720 images are captured. The workflow for OPT image reconstruction is described in Appendix B. Brightfield OPT (Figure 5a) allowed distinguishing regions with increased light absorption, such as pigmentation along all the tissue and the egg mass. Moreover, it was possible to observe the adult female and giant cells. In fluorescence OPT (Figure 5b), a strong auto-fluorescence signal was detected on the egg masses and the characteristic shapes of the adult female and the giant cells were also visible. 

## 3. Discussion

The main goal of this work was to fine-tune clearing and microscopic methods to facilitate deep-tissue imaging of intact plant roots in order to visualize both morphological changes in root tissues and the development stage of the nematode derived from the nematode–plant interaction. Tomato cv. Durinta and eggplant cv. Cristal infected with *M. incognita* were employed to determine whether reducing the differences in refractive indices within the tissue improved the optical transparency of biological samples. In a previous work of the group [5], RKN infected samples were cleared with ethanol–BABB. Here we propose another solvent-based clearing alternative, ECi clearing, that is simple, cheap and, overall, non-toxic or corrosive. We also present simplified clearing protocols that do not need fixation steps since, in our hands, auto-fluorescence was naturally preserved from endogenous fluorophores, and not induced by glutaraldehyde fixation as reported in [32,33].

In addition, we tested the effect of different dehydration solvents (ethanol, methanol or 1-propanol) in both ECi and BABB protocols, to efficiently render tomato and eggplant roots optically transparent, since it has been observed that they affect the outcomes. During the clarification process, we proved that the formulations developed for tissue clearing are effective at clearing roots. However, the two different species studied show different behavior. We observed that the results are more favorable in tomato roots than in eggplant roots, suggesting the existence of differences in the tissue composition of the two specimens tested. Furthermore, in terms of the effectiveness of dehydration with alcohols, the immersion of tomato roots in 1-propanol produces the best results, especially combined with ECi. In the case of eggplant, the most favorable combination is ethanol–BABB. It has also been observed that ECi causes slight shrinkage of the tissue during the transparency process, in any case, smaller than when using BABB. These results show that clarification methods need to be tuned to each of the analyzed crop species. 

From the microscopic point of view, in the literature, 3D visualization of nematode–root interaction has normally been performed using confocal microscopy. However, it presents several limitations. Here we present two alternatives to confocal microscopy: light sheet microscopy and optical projection tomography. Both systems can be easily integrated in the same setup [29,30,31]. The major advantage of LSFM/OPT for the study of a root’s anatomy is the possibility of obtaining fully volumetric reconstructions of the specimen under study. This is due to the microscope architecture, with an orthogonal illumination–detection scheme, and the sample mounting procedure suspended between the objectives. For this reason, samples are free to rotate, providing different views that can be computationally fused into a single dataset (Figures S1 and S2). Conversely, confocal microscopy is restricted to a single view. Compared to confocal microscopy, LSFM and OPT allow bigger areas of the sample to be imaged with a similar resolution in a reduced lapse of time. Additionally, the use of sCMOS cameras, instead of photomultiplier tubes (PMT), allows the detection of weaker auto-fluorescence signals.

Although OPT and LSFM share the ability to rotate the sample and can be mounted on the same equipment, they have several distinct features, rendering both of them optimal for different imaging tasks. LSFM provides a higher lateral resolution (x and y), but a lower axial resolution (z) compared to OPT because the point spread function (PSF) is elongated. This results in non-isotropic voxels, which may give rise to ambiguities for 3D analysis, while, due to its respective detection principle, OPT does provide true isotropic spatial resolution (i.e., voxels with identical dimensions along the x, y and z-axes). Therefore, OPT is more suitable for carrying out measurements because the pixels have the same measurement in all directions. Although LSFM image acquisition takes longer than OPT, 3D volume rendering is faster than OPT since a reconstruction method is not required. However, storage and handling of the huge amount of data produced by LSFM pose significant challenges. In contrast, OPT greatly improves imaging speed, and the amount of data produced is reduced, but post-acquisition data processing is more tedious. The pros and cons of each technique are summarized in Supplementary Table S1.

Finally, but not less important, these technologies have become, during the last year, a cheap alternative to high-end commercial microscopes thanks to several open source initiatives, such as OpenSPIM [34], OpenSpinMicroscopy [29], Legolish [35] or other 3D printing approaches [36]. These platforms allow not only democratizing this technology, but also to foster its adoption for non-experienced laboratories. In general, the cost of a basic LSFM system, such as the one presented here, is at least an order of magnitude lower than confocal microscopes. OPT is even cheaper, only requiring an LED, a camera and a rotation motor. 

In conclusion, here we present LSFM and OPT as a cheap and affordable alternative to confocal microscopy for nematode–root interaction characterization by means of auto-fluorescence signal. The improvement of optical penetration deep into the whole sample by the treatment with ECi suggests that it is possible to investigate the different stages of the life cycle of *M. incognita* inside the specimen, due to the observation of nematodes, eggs masses and giant cells within root galls. The results show that the proposed protocol that uses 1-propanol as a dehydration agent and ECi as an RI-matching reagent is the most suitable for imaging tomato roots. However, for imaging eggplant roots, the most favorable combination is still ethanol–BABB, although 1-propanol–ECi is also suitable. The methodology here presented can be extended to other species, and successful results have already been obtained in different sorghum, mustard and radish varieties, providing a framework for the study of the degree of infection, plant resistance or nematode trapping. Moreover, this microscopic technique can also be used to study the dynamics of other plant–nematode interactions along with the anatomical alterations in plant tissue caused by them [37].

## 4. Materials and Methods

### 4.1. Plant Material and Nematode Inoculums

Seeds of the tomato cv. Durinta and eggplant cv. Cristal, both susceptible to *M. incognita* [38,39], were sown into vermiculite, maintained in a growth chamber at 25 ± 2 °C and 16:8 h light–dark photoperiod, irrigated as needed and fertilized with Hoagland solution once a week. At three-leaf stage, plants were transferred to 200 cm^3^ pots filled with sterilized sand (121 °C for 1 h and repeated after 1 day) and maintained in the same conditions previously stated. 

Second-stage juveniles of the *Meloidogyne incognita* were used as inoculums. Nematode eggs were extracted from tomato roots by blender maceration in a 40 g/L of NaOCl solution for 5 min [40]. Then, the suspension was passed through a 74 µm aperture sieve to remove root debris. The eggs were collected on a 25 µm aperture sieve and placed on Baermann trays [41]. The J2 that emerged during the first 24 h were discarded. Afterwards, J2 were collected daily for a week and were kept at 9 °C until inoculation.

The plants were inoculated 5 days post-transplantation at a rate of 1 J2/cm^3^ of soil applied into two opposing holes 3 cm deep and 4 cm apart, which were covered with soil, and maintained in the same conditions described previously for 6 weeks.

### 4.2. Sample Preparation

For all sample preparations, tomato and eggplant roots were first rinsed in running water and then cut to a maximum size of approximately 4 cm in a long way. Root galls were transferred sequentially into a dehydration series of 25%, 50%, 75% and 100% dehydration agents. For comparison of dehydration agents, three different agents were exchanged: 1-propanol, ethanol and methanol, respectively. The dehydration process was performed for at least 24 h per dehydration step in 14 mL polypropylene Falcon tubes containing 5 mL dehydration agent at room temperature. After dehydration, roots were transferred in a 14 mL tube with at least 5 mL of clearing reagent and incubated at room temperature until they became transparent. For comparison of clearing reagent, clearing protocol was performed via either BABB (1 part benzyl alcohol and 2 parts benzyl benzoate) or ECi (ethyl cinnamate). The samples were checked at daily intervals until the desired degree of transparency was achieved and recordings were acquired over the following days.

### 4.3. Experimental Setup

A home-made system, combining LSFM and OPT imaging, was specifically developed for this study. More details can be found in Appendix A.

### 4.4. Image Post-Processing

For LSFM and OPT image reconstruction, the open source image processing software FIJI (Fiji Is Just ImageJ) [28], based on ImageJ2, was used. In the case of OPT images, the raw data were processed using the Fiji’s OptiJ plugin [42], as described in Appendix B. OPT 3D reconstruction are visualized using the Fiji’s open source volume-render plugin ClearVolume [43]. 

## Figures and Tables

**Figure A1:**
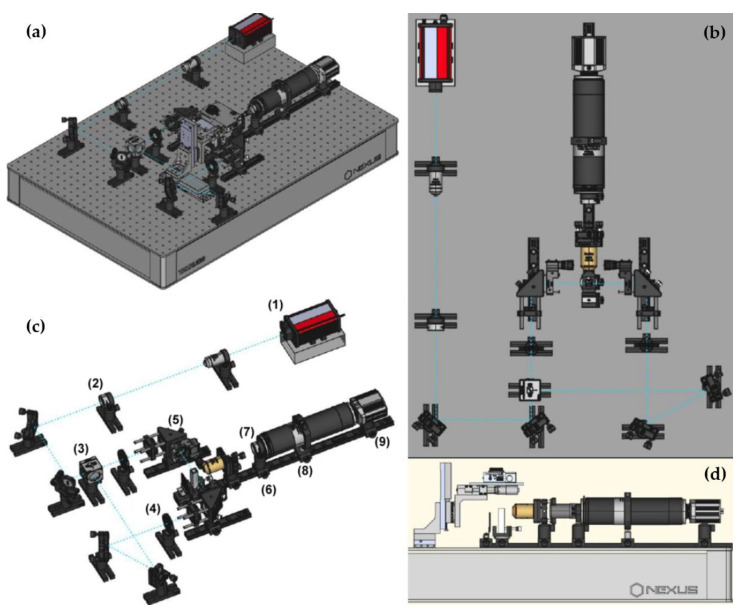
Schematic representation of the experimental microscope setup. (**a**) Three-dimensional representation of LSFM system (isometric view). (**b**) Three-dimensional representation of the LSFM system without the sample mounting and monitoring system (isometric view). ((1) laser, (2) beam expander, (3) beam splitter, (4) pinholes, (5) cylindrical lens, (6) filters, (7) iris diaphragm, (8) tube lens, (9) camera.) (**c**) Top view of the path followed by the laser light beam. (**d**) Representation of the OPT system (lateral view). Drawings were generated using FreeCAD open source program.

**Figure A2:**
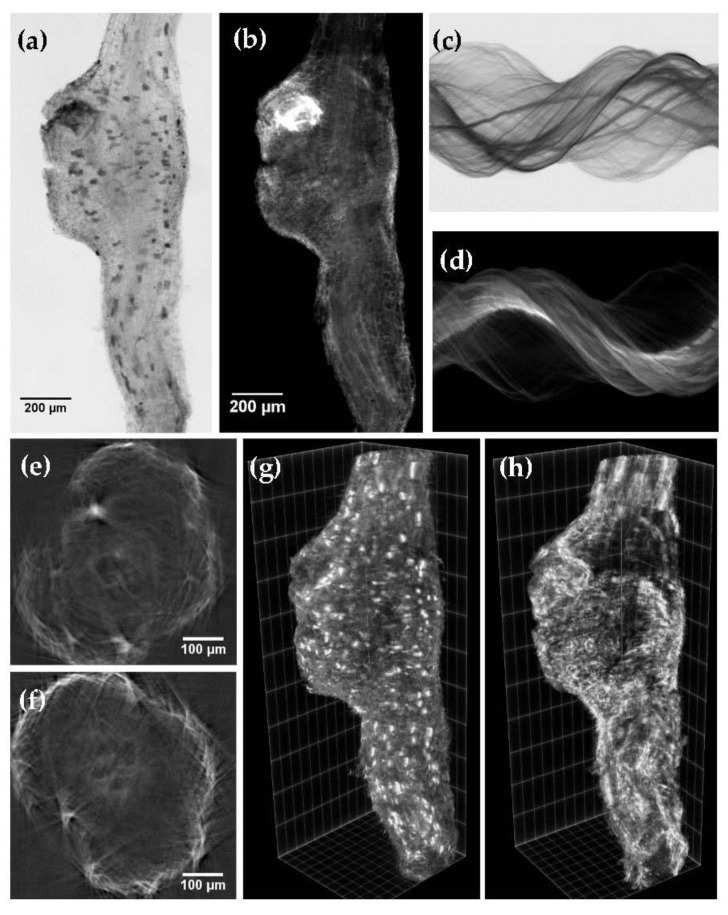
OPT raw data images of tomato roots induced by *M. incognita* 6 weeks after inoculation cleared with 1-propanol–ECi in (**a**) brightfield and (**b**) fluorescence mode. Scale bar: 200 µm. (**c**) Brightfield sinogram and (**d**) fluorescence sinogram. (**e**) Brightfield OPT stack reconstruction and (**f**) fluorescence OPT stack reconstruction, after back-projection algorithm. Scale bar: 100 µm. Three-dimensional reconstructions of roots in (**g**) brightfield and (**h**) fluorescence modes.

## Data Availability

Request for materials should be addressed to E.J.G.

## Supplementary Materials

The following supporting information can be downloaded at: https://www.mdpi.com/article/10.3390/plants11091165/s1, Figure S1: LSFM cross-section of tomato root gall cleared with 1-propanol–ECi protocol, recorded at different view angles. Figure S2: LSFM cross-section of eggplant root gall cleared with ethanol–BABB and 1-propanol–ECi protocol, recorded at different view angles. Figure S3: Photographs of the custom-made LSFM/OPT system. Figure S4: Front panel of the custom-made LabView software for LSFM/OPT image acquisition. Table S1: Comparison of characteristics between LSFM and OPT. Video S1: LSFM 3D stack of a tomato root cleared with 1-propanol–ECi protocol (single color). Video S2: Two color LSFM 3D stack of a tomato root cleared with 1-propanol–ECi protocol. Video S3: Two color LSFM 3D stack of a tomato root cleared with ethanol–BABB protocol. Video S4: Two color LSFM 3D stack of a tomato root cleared with 1-propanol–BABB protocol. Video S5: Two color LSFM 3D stack of an eggplant root cleared with ethanol–BABB protocol. Video S6: Two color LSFM 3D stack of an eggplant root cleared with 1-propanol–ECi protocol. Video S7: Two color LSFM 3D stack of an eggplant root cleared with methanol–BABB protocol. Video S8: Two color LSFM 3D stack of an eggplant root cleared with 1-propanol–BABB protocol. Video S9: OPT imaging of a tomato root cleared with 1-propanol–ECi protocol, in brightfield and fluorescence modes.

## Appendix A

Here we describe the details of the custom LSFM/OPT system developed for this work (see also Figure S3).

The microscope is equipped with a modulated laser diode source with a wavelength of 488 nm (60 mW, Cobolt 06-MLD 488 nm). In the illumination path, the laser beam is expanded using a telescope system composed of a microscope objective lens (Hertel & Reuss, 40× numerical aperture (NA): 0.65) and an achromatic lens (AC254-200-A, Thorlabs, Newton, NJ, USA). A beam splitter (BS019, Thorlabs, Newton, NJ, USA) is used to illuminate the sample more homogeneously from both sides. These identical illumination units are composed of a cylindrical lens (f = 150 mm, LJ1629RM, Thorlabs, Newton, NJ, USA), which generates the light sheet, mounted in a rotation mount (CRM1LT, Thorlabs, Newton, NJ, USA) and a right-angle kinematic mirror (KCB1E, Thorlabs, Newton, NJ, USA). Before each cylindrical lens, a pinhole allows adjusting the light sheet thickness and the illuminated field of view (FoV).

In the detection path, the fluorescence signal is collected by a 4× Plan Fluor Nikon detection objective with a 17.20 mm working distance and 0.13 NA. Laser light is filtered out using different filters (525/45 nm (green filter), 561/LP nm (yellow filter) or 650/LP (red filter)), depending on the experiment. After the filter, and in front of an infinity-corrected tube lens (TTL200-A, Thorlabs, Newton, NJ, USA), a lever-actuated iris diaphragm (SM2D25, Thorlabs, Newton, NJ, USA) allows extending the depth of field (DoF) of the system. The collected fluorescence signal is imaged onto an ORCA-Flash4.0 V3 Digital CMOS camera (Hamamatsu, Japan) with 6.5 × 6.5 μm^2^ pixels.

The sample positioning system consists of two DC servo motorized translation stages (MTS25-Z8 and MTS50-Z8, Thorlabs, Newton, NJ, USA) for sample lateral and vertical positioning and a stepper motor rotation mount (K10CR1/M, Thorlabs, Newton, NJ, USA) for sample rotation. Sample scanning for LSFM is performed with a stepper motor translation stage (LNR25ZFS(/M), Thorlabs, Newton, NJ, USA). A 45 × 12.50 × 12.5 mm glass cuvette (Z803081-1EA, Hellma, Müllheim, Germany) is used as the immersion chamber for the samples during imaging. This is filled with the mounting medium (BABB or ECi) depending on the protocol. Samples are assembled into a custom-made sample holder using the structure of a syringe and a lead dispenser system of a mechanical pencil mounted on a kinematic pitch/yaw adapter (KAD8F, Thorlabs, Newton, NJ, USA) (Figure S3). To complement the setup, an elemental brightfield illumination arm is placed for sample visualization and alignment, using a white light-emitting diode (LED) (MEBL-CW25, Moritex, Saitama, Japan). The whole detection system is carefully aligned and mounted on a rail that allows the exchange of parts to be a simpler process.

For image acquisition, home-made software developed in LabVIEW (Laboratory Virtual Instrument Engineering Workbench) was used (Figure S4). The software allows controlling the laser and LED ON/OFF system, the sample movement through rotation and translation motors, and the data acquisition and image display from the camera.

## Appendix B

To reconstruct a 3D volume from OPT projections through a filtered back-projection algorithm, corrected projection stacks require data processing. In that direction, the Fiji [28] plugin OptiJ [42] is used to perform the 3D reconstruction. Following the workflow, the raw data projection stack (Figure A2a,b) is processed into a sinogram (Figure A2c,d), an intermediate step in the reconstruction. Then, the sinogram is processed to obtain the 2D stack reconstruction (Figure A2e,f). From the 2D reconstruction, the reconstructed slices can be stacked resulting in a 3D reconstruction of the imaged sample. Finally, volumetric reconstruction is displayed using the Fiji plugin ClearVolume [43] (Figure A2g,h). To remove artifactual streaks that degrade image quality and worsen the reconstruction resolution of the sample, we used the “bleach correction” command, included in the ImageJ/Fiji library.
