# Supplementary material for: Development of Microscopic Techniques for the Visualization of Plant–Root-Knot Nematode Interaction
Source: Plants (Basel). 2022 Apr 26;11(9):1165. doi: 10.3390/plants11091165 (PMC9104198; doi:10.3390/plants11091165)
Supplement: Supplementary file 1 [file plants-11-01165-s001.zip › Supplementary Material/Supplementary_Material.pdf]

Development of microscopic techniques for the visualization of plant-root-knot nematode interaction

Helena Vernet, Aïda Magdalena Fullana, F. Javier Sorribas and Emilio J. Gualda

<u>Supplementary Figures and Tables</u>

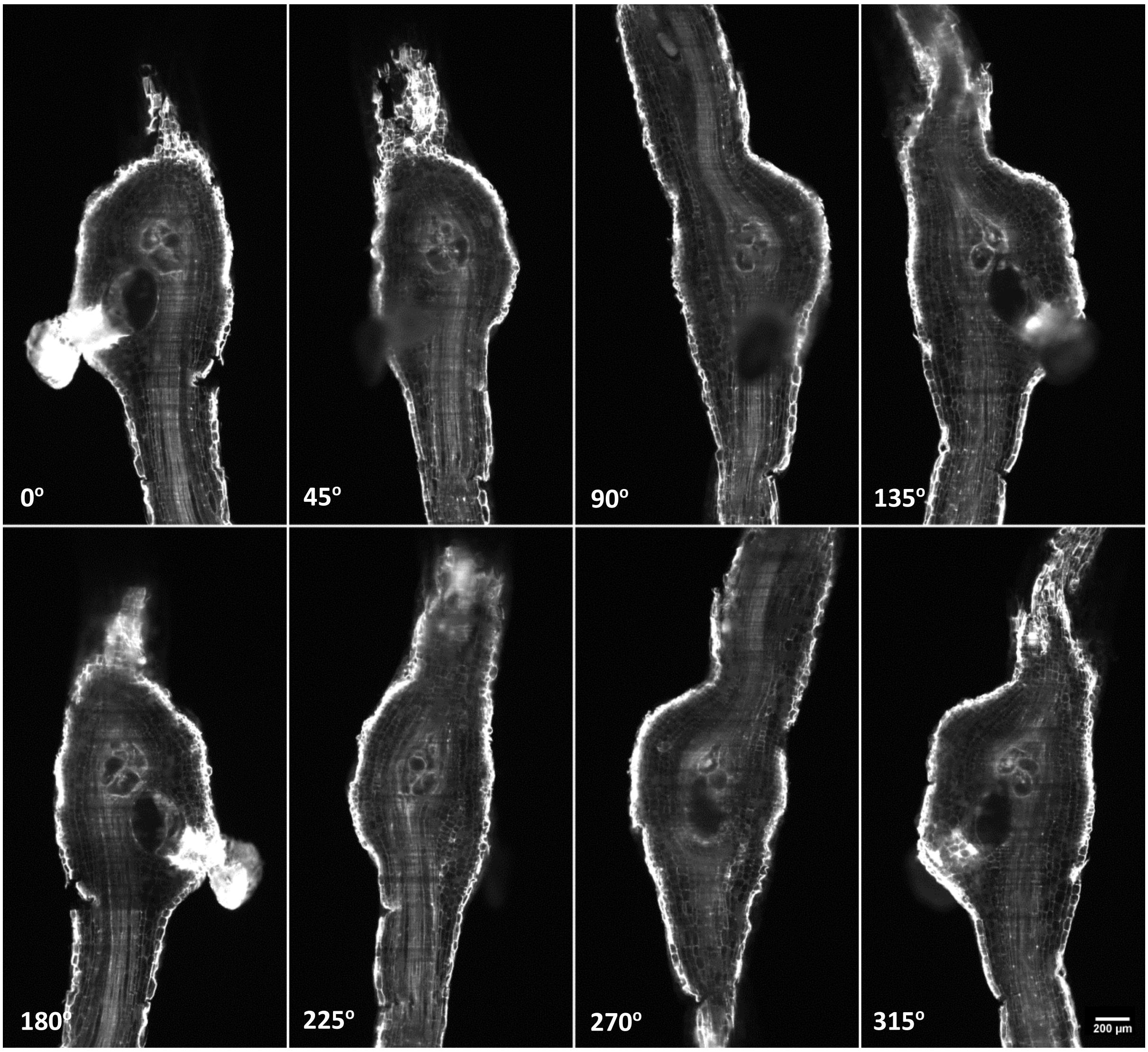

Figure S1. LSFM cross-section of tomato root galls cleared with to 1-propanol –ECi protocol, recorded at different view angles. Scale bar: 200 µm.

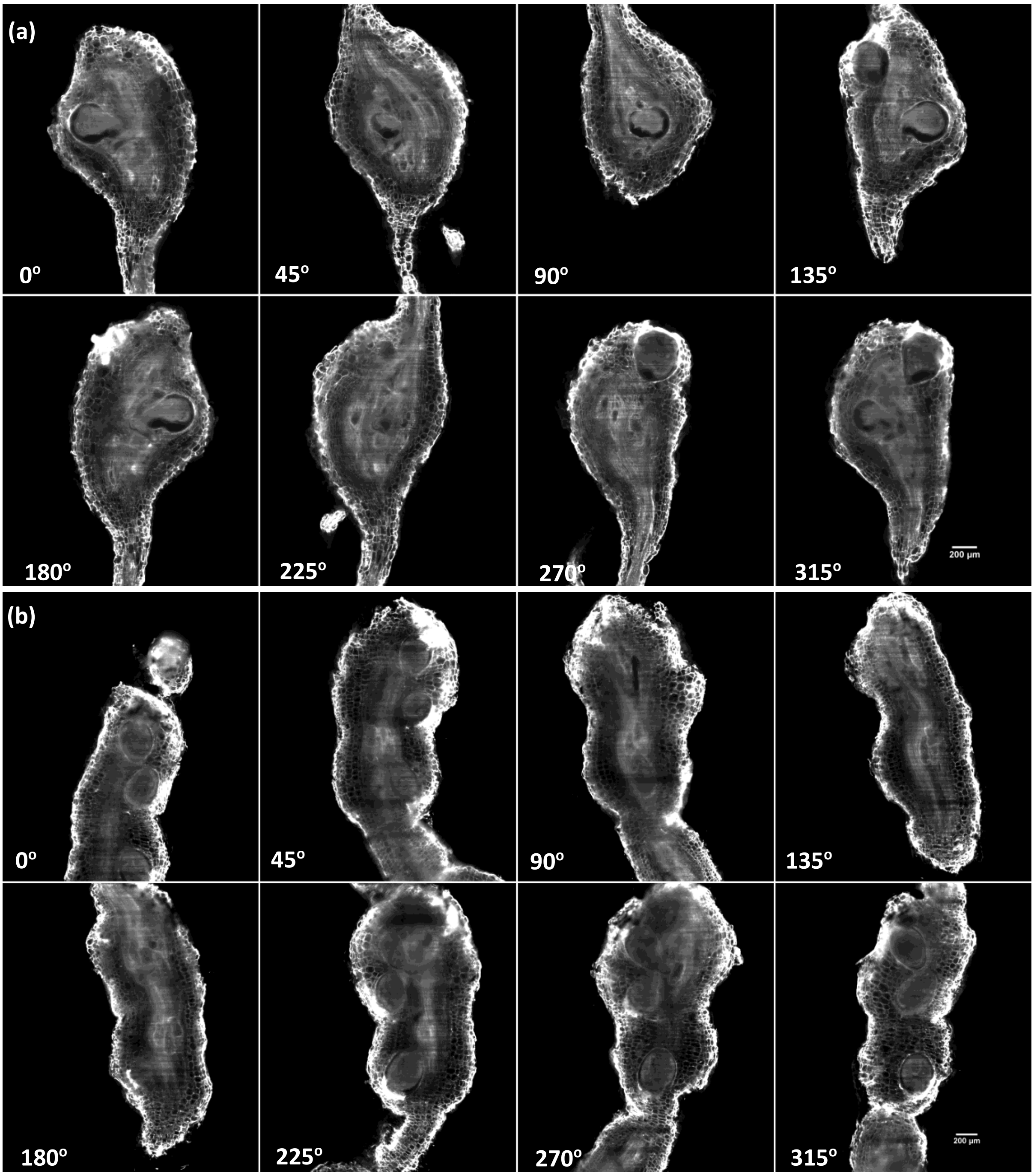

Figure S2. (**a**) LSFM cross-section of eggplant root galls cleared with to ethanol–BABB protocol, recorded at different view angles. (**b**) LSFM cross-section of eggplant root galls cleared with to 1-propanol –ECi protocol, recorded at different view angles. Scale bar: 200 μm.

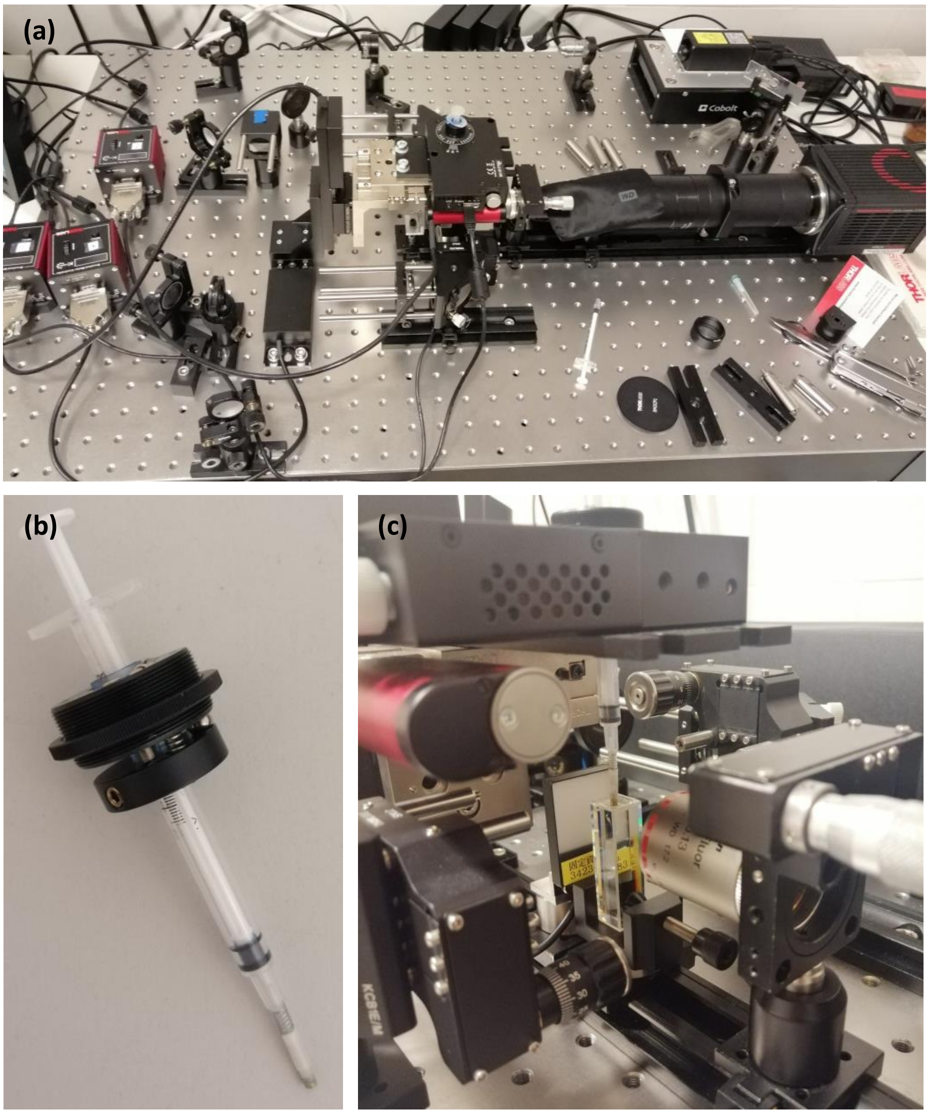

Figure S3. (**a**) Photograph of the custom made LSFM/OPT system (**b**) Photograph of the sample mounting system using a kinematic pitch/yaw adapter (KAD8F, Thorlabs**).** (**c**) Photograph of a mounted sample in a cuvette.

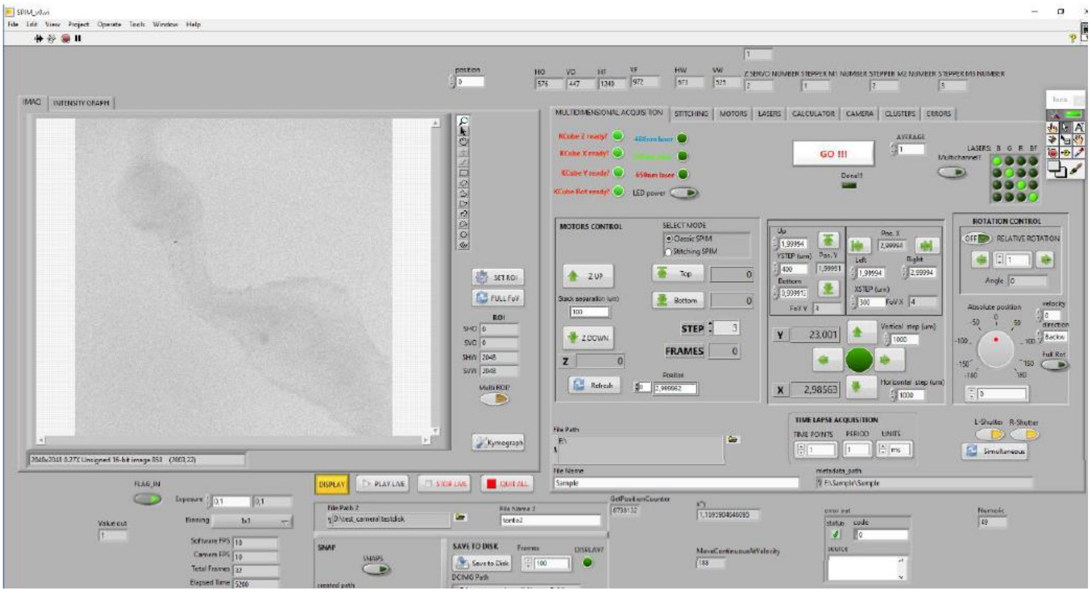

Figure S4. Front panel of the custom made LabView software for LSFM/OPT image acquisition.

	LSFM	OPT
Resolution	High lateral resolution (x and y), low axial resolution (z)	Low resolution, but provide isotropic spatial resolution (voxels with identical dimensions along the x, y, and z axes)
Acquisition time	Slow (sequential view acquisition)<br>5-7 minutes	Fast (depending on the rotation program chosen)<br>3 minutes on the continuous method and 15-20 on the step-by-step method.
Post-processing time	Computational post-processing is not required	Computational post-processing is required. With the full frame, it can take between 24 and 48 hours. For this reason, it is customary to reduce to 8 bits to process in less time and we use binned data. But this leads to a loss of resolution and image quality.
Data volume	Huge amount of data.<br>An average of to 10 GB for each stack acquisition. (2048x2048px/16bits)	Less amount of data.<br>An average of 1.44 GB (1024x1024 px) (Or 5.6 GB (2048x2048px)) for each stack acquisition, and 114 MB for each 2D reconstructed stack.

Table S1. Comparison of characteristics between LSFM and OPT
